# An Ensemble Deep Learning based Predictor for Simultaneously Identifying Protein Ubiquitylation and SUMOylation Sites

**DOI:** 10.1186/s12859-021-04445-5

**Published:** 2021-10-24

**Authors:** Fei He, Jingyi Li, Rui Wang, Xiaowei Zhao, Ye Han

**Affiliations:** 1 School of Information Science and Technology, Northeast Normal University, Changchun, 130117 China; 2 Key Laboratory of Symbolic Computation and Knowledge Engineering of Ministry of Education, Jilin University, Changchun, China; 3 School of Information Technology, Jilin Agricultural University, Changchun, China

**Keywords:** Protein ubiquitylation site, Protein SUMOylation site, Convolution neural network, Deep learning, Ensemble learning

## Abstract

**Background:**

Several computational tools for predicting protein Ubiquitylation and SUMOylation sites have been proposed to study their regulatory roles in gene location, gene expression, and genome replication. However, existing methods generally rely on feature engineering, and ignore the natural similarity between the two types of protein translational modification. This study is the first all-in-one deep network to predict protein Ubiquitylation and SUMOylation sites from protein sequences as well as their crosstalk sites simultaneously. Our deep learning architecture integrates several meta classifiers that apply deep neural networks to protein sequence information and physico-chemical properties, which were trained on multi-label classification mode for simultaneously identifying protein Ubiquitylation and SUMOylation as well as their crosstalk sites.

**Results:**

The promising AUCs of our method on Ubiquitylation, SUMOylation and crosstalk sites achieved 0.838, 0.888, and 0.862 respectively on tenfold cross-validation. The corresponding APs reached 0.683, 0.804 and 0.552, which also validated our effectiveness.

**Conclusions:**

The proposed architecture managed to classify ubiquitylated and SUMOylated lysine residues along with their crosstalk sites, and outperformed other well-known Ubiquitylation and SUMOylation site prediction tools.

## Background

Ubiquitin [1, 2] is a small protein composed of 76 amino acids in eukaryotes. Through the catalytic action of activating enzyme (E1), binding enzyme (E2), and ligase (E3) [3, 4], ubiquitins can covalently connect to the lysine residues of the target proteins [5, 6]. As a major member of the family, small ubiquitin-related modifier (SUMO) proteins have similar 3D structures and biological modification processes to ubiquitins [7, 8]. They are both highly conserved in evolution and related to diverse cellular activities including gene location, gene expression, and genome replication [9]. However, numerous potential Ubiquitylation and SUMOylation sites remain to be discovered from protein sequences.

Since most ubiquitinated and SUMOylated proteins are short-lived proteins with poor stability, the experimental approaches to identify protein Ubiquitylation and SUMOlytion sites might be costly and time-consuming [10]. Therefore, it is worthwhile to study the computational approaches.

At present, several sequence-based approaches have been proposed to carry out the prediction of protein Ubiquitylation and SUMOylation sites respectively. Huang et al. [11] developed a method called UbiSite, using an efficient radial basis function (RBF) network to identify protein Ubiquitylation sites. Next, Chen et al. [12] established UbiProber, which extracted a set of features including physico-chemical property (PCP) and amino acid composition(AAC) to make Ubiquitylation site prediction. Subsequently, Radivojac et al. [13] proposed a random-forest based predictor UbPred, in which 586 sequence attributes were detected from the input features. GPS-sumo [14] employed a group-based prediction system (GPS) by a similarity clustering strategy to identify SUMOlytion sites. JASSA by Guillaume et al. [15] uses a scoring system based on a position frequency matrix. Then, pSumo-cd [16] applied a covariance discriminant algorithm in combination with a pseudo amino acid composition model. A recent work HseSUMO [17] only employed four half-sphere exposure-based features to predict SUMOylation sites. In addition to the individual prediction of Ubiquitylation or SUMOylation sites, mUSP was proposed to predict their crosstalk. They treated these three types as three binary problems independently. However, these traditional machine learning methods employed feature engineering, which may lead to incomplete representations and biased results.

Deep learning as a cutting-edge representation learning technique enables the production of high-level semantic features without handcrafted design [18], it has been widely applied to several PTM problems with large datasets [19, 20]. Wang et al. [21, 22] proposed a deep learning predictor MusiteDeep, based on convolutional neural networks, to predict and visualize protein post translational modification sites. Chen et al. [23] built a computation model, MUscADEL, based on the long short term memory (LSTM) recurrent neural network. Fu et al. [24] used Matlab to implement deepUbi, a protein Ubiquitylation site prediction tool. Due to that its backend Matlab is a closed commercial software, its availability is limited. Although deep learning has been applied to PTM problems, the similarities between the two PTMs have not been recognized or fully exploited. To our best knowledge, there is no site prediction tool based on deep learning to predict protein Ubiquitylation and SUMOylation sites simultaneously.

In this paper, we proposed an ensemble deep learning based predictor for identifying protein Ubiquitylation and SUMOylation sites as well as their crosstalk sites simultaneously. The ensemble learning layer integrated different types of physico-chemical properties of amino acids. The network can learn the high-level representation from the raw protein sequence and its corresponding physico-chemical properties. Owing to the similarity of biochemical processes of Ubiquitylation and SUMOylation, the Ubiquitylation and SUMOylation datasets were used for training simultaneously, which not only circumvents the scarcity of training data but also endows the model with more discerning power.

## Result

### Overview

**Fig. 1:**
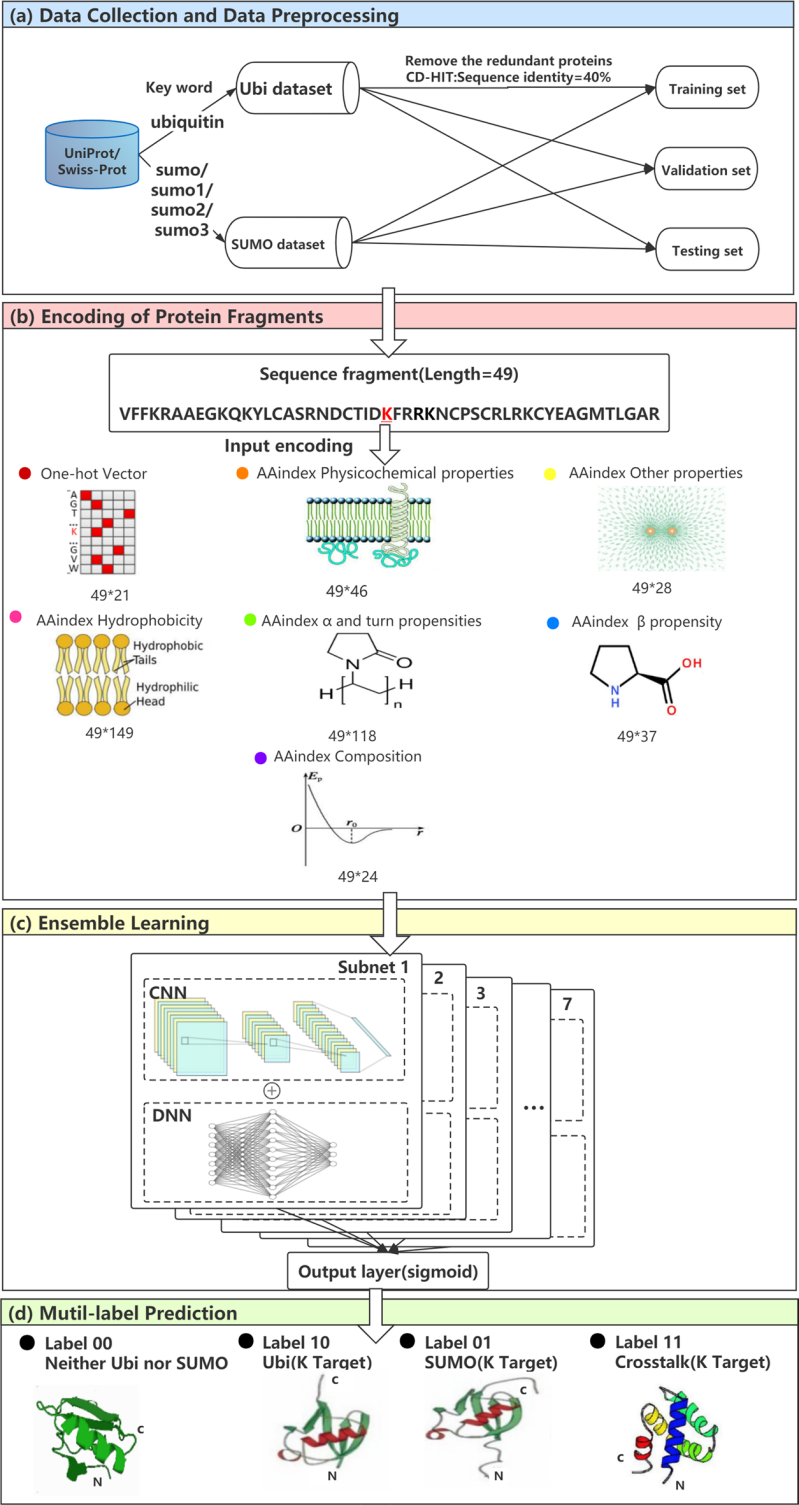
The overview of our workflow for predicting protein Ubiquitylation and SUMOylation sites. **a** Data collection and preprocessing of Ubiquitylation and SUMOylation sites. **b** Encode fragments of proteins and input seven networks. **c** The deep network architecture adopts CNN and DNN. The output layer integrated the prediction results of the seven subnets. **d** The output contains four types of sites: negative, Ubiquitylation site, SUMOylation site and Crosstalk site

**Fig. 2:**
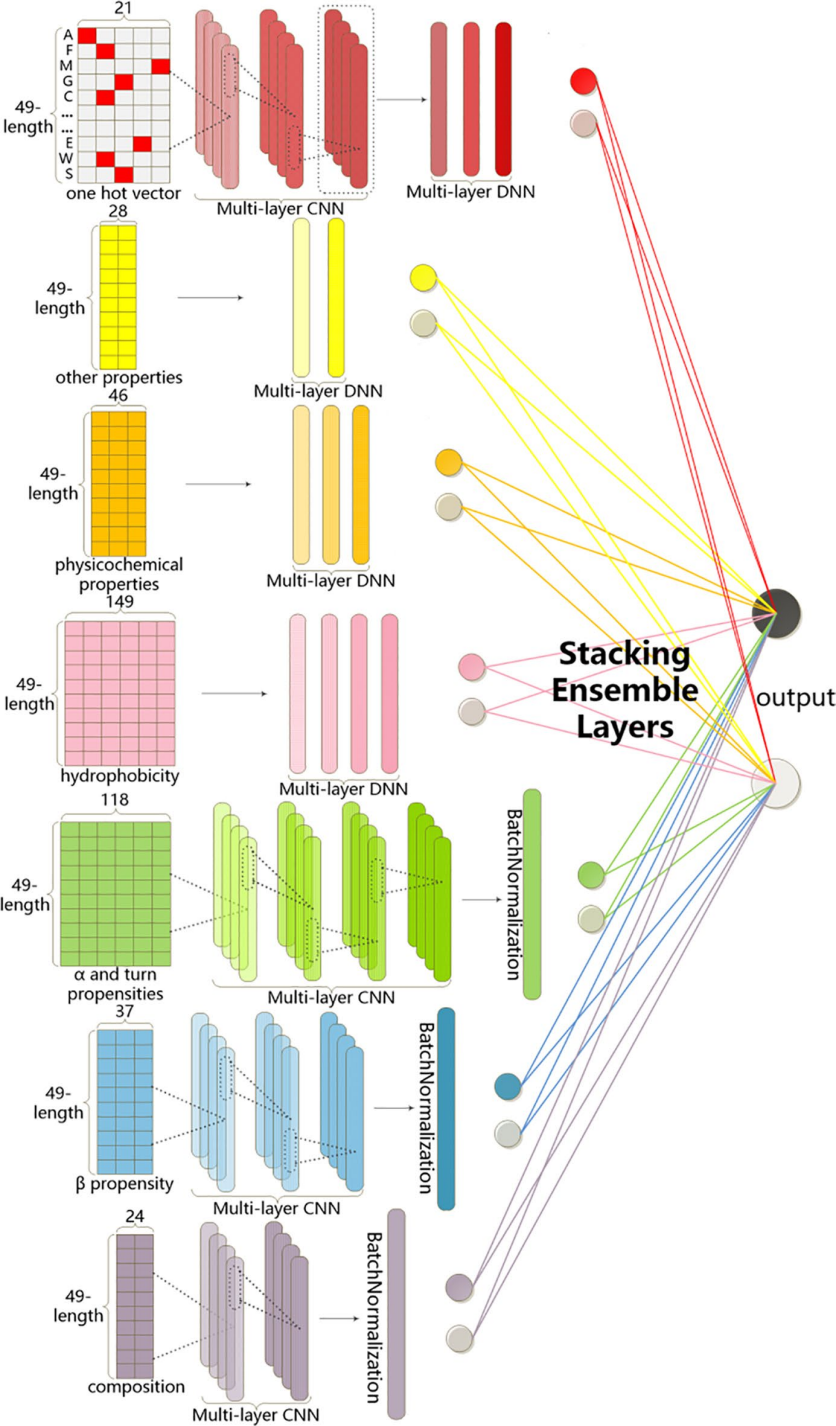
Our proposed deep architecture. The architecture consisted of seven subnets to separately handle one-hot and six physical-chemical properties, and then their detective high-level representation into an ensemble layer to predict final results

Figure 1 provides an overview of our workflow. We formulated the process of protein Ubiquitylation and SUMOylation site prediction as a multi-label classification problem. First, we collected the protein sequences of Ubiquitylation and SUMOylation from UniProt/Swiss-Prot, used CD-HIT to remove the redundant sequences that have more than 40$\%$ sequence identity, and split the remaining data into the training set, validation set, and testing set. Next, all the fragments of sequences were encoded and inputted into seven respective deep networks. Then, we proposed an ensemble learning layer to integrate multiple protein representations as shown in Fig. 2. It integrates seven supervised learning subnets, each of which utilized convolution layers or fully connected layers, to extract deep representations from protein sequence features. At last, since we targeted two categories for multi-label classification, we defined the output of our tool using dummy code, in which Ubiquitylation and SUMOylation sites independently associated with different labels. The 2-dimensional code 10 was set to represent Ubiquitylation sites and 01 was assigned to SUMOylation sites, while code 11 denoted the crosstalk (both Ubiquitylation and SUMOylation) sites and code 00 was encoded for negatives. The output layer of our deep model was set to 2 neurons to generate multi-label results by using the sigmoid [25] activation function, which independently produced a probability for each category.

### Comparative results among the ensemble model and seven meta classifiers

**Fig. 3:**
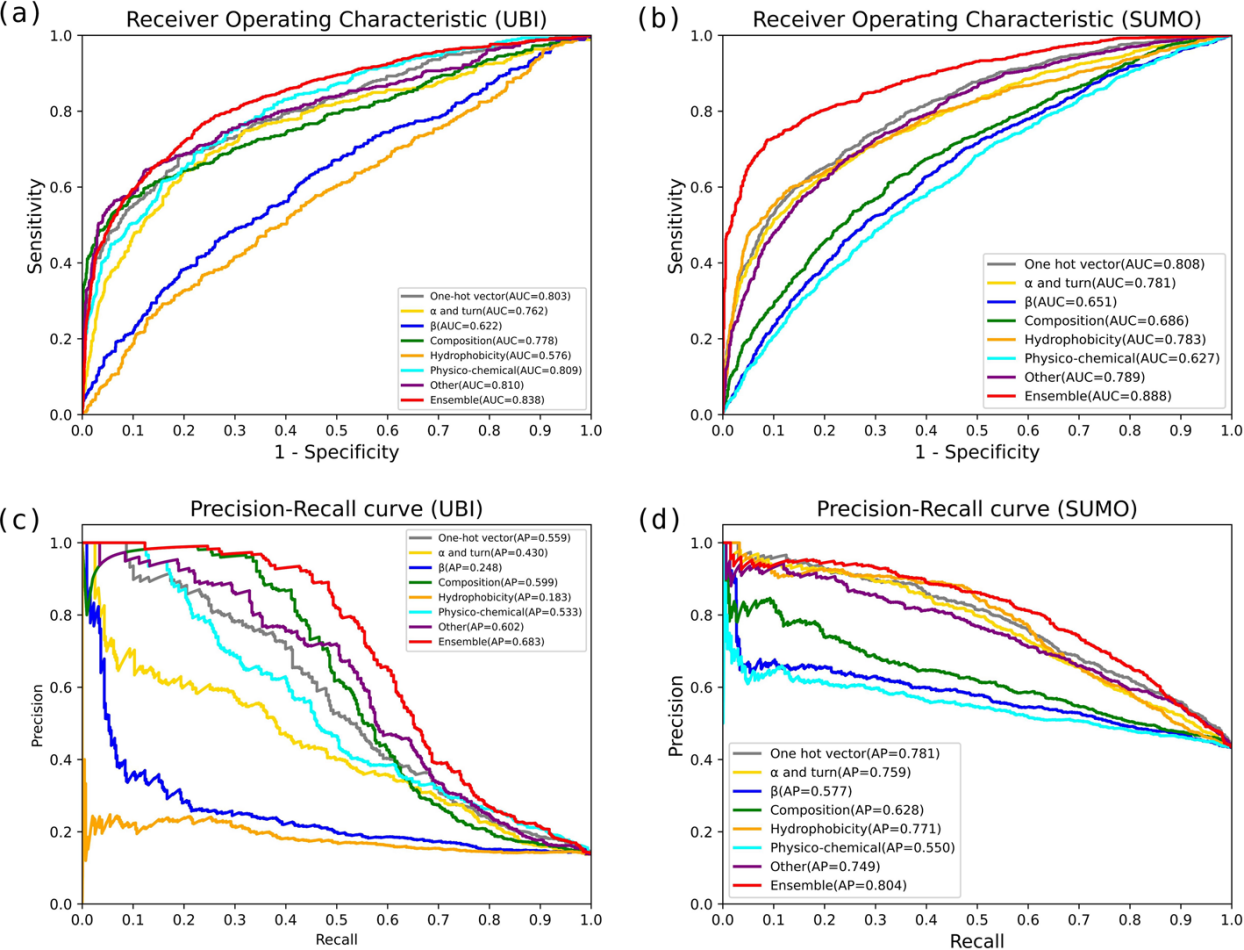
Comparisons between our ensemble network and seven subnets ROC curves on **a** Ubiquitylation site prediction and **b** SUMOylation site prediction, and PR curves on **c** Ubiquitylation site prediction, and **d** SUMOylation site prediction

We compare between the ensemble model and seven meta classifiers to provide deeper insight into the advantage of our ensemble learning strategy. As we have pretrained each meta subnet before integrating to the ensemble architecture, the performance of the meta classifiers can be easily assessed on the same test data by loading pretrained weights. The comparative results are shown in Fig. 3.

From this figure, we can observe the meta classifiers showed varying degrees of effectiveness, and offered deep representations from different perspectives. Such meta classifiers with sufficient precision and diversity provided a good ensemble foundation. In addition, these meta classifiers performed differently between protein Ubiquitylation and SUMOylation sites. For instance, the physico-chemical subnet performed top-3 rank out of the 7 meta classifiers on Ubiquitylation sites while it ranked as last of the 7 meta-classifiers on SUMOylation sites. This demonstrated how an adaptive ensemble was required to properly combine all meta classifiers for different categories.

### Results of protein Ubiquitylation and SUMOylation sites prediction

**Fig. 4:**
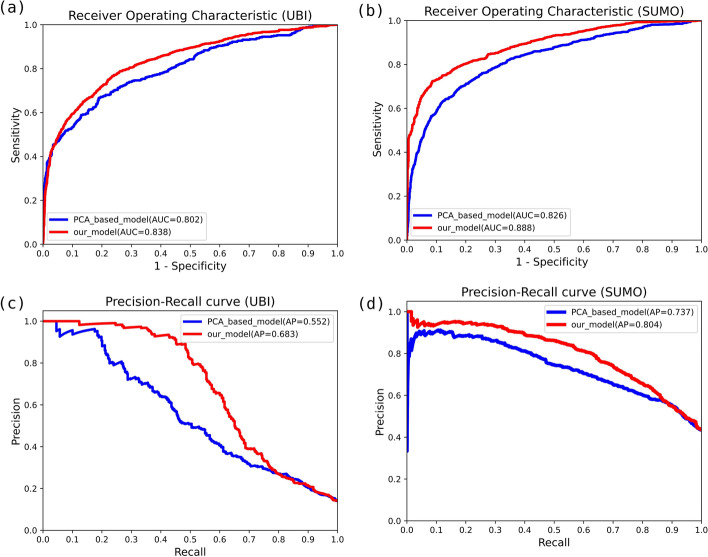
Comparisons between our method and others on our test set. The performance of the multi-label model with ROC curves on **a** ubiquitylation site prediction and **b** SUMOylation site prediction, and PR curves on **c** ubiquitylation site prediction, and **d** SUMOylation site prediction. The red line represents the average result of the tenfold cross-validation for our tool. The dashed lines and marks in other colors represent the results of popular Ubiquitylation and SUMOylation site prediction tools. The reason we used marks here is that those tools returned classification results instead of predictive probabilities. We are unable to plot ROC and PR curves with the predictive probabilities

We compared our method with several popular and accessible protein ubiquitination and SUMOylation site prediction tools (Ubisite [11], Ubiprober [12], Ubpred [13], psumo-cd [16], JASSA [15], sumoplot [26], GPSsumo [14], and MUscADEL) [23] by submitting our testing dataset to their websites. Their performance was plotted as ROC and PR curves in Fig. 4. Our AUC values were 0.838 on Ubiquitylation site prediction and 0.888 on SUMOylation site prediction respectively. A similar situation appeared on the PR curves, where the AP value of Ubiquitylation site prediction was 0.683 and the AP value of SUMOylation site prediction was 0.804. As shown in Fig. 4, the performance of the proposed deep learning architecture was superior to other protein Ubiquitylation and SUMOylation site prediction tools for each measure.

**Fig. 5:**
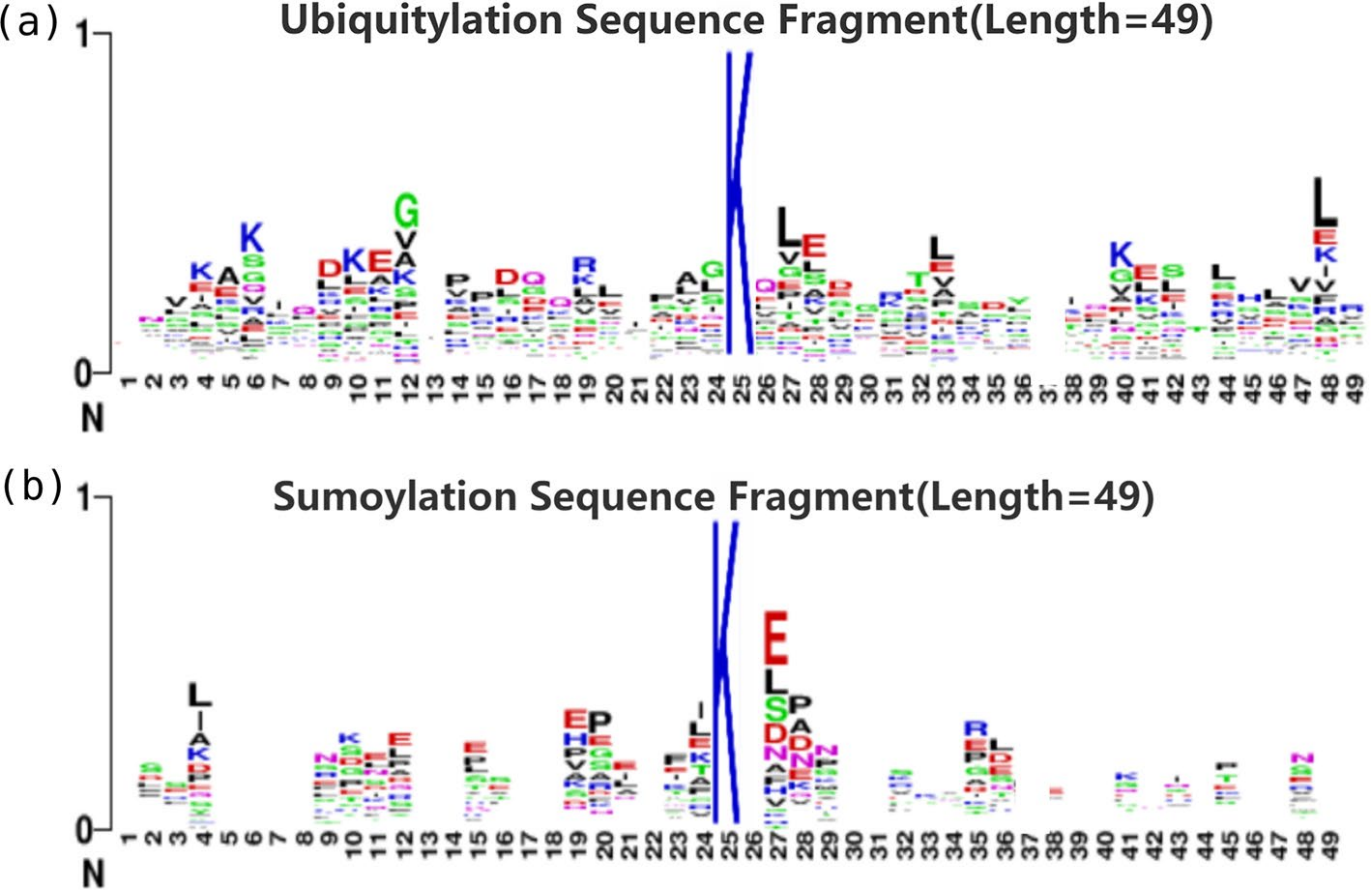
WebLogo visualization of the position-specific amino acid composition at **a** difference between upstream and downstream fragments around the Ubiquitylation sites **b** difference between upstream and downstream fragments around the SUMOylation sites

**Fig. 6:**
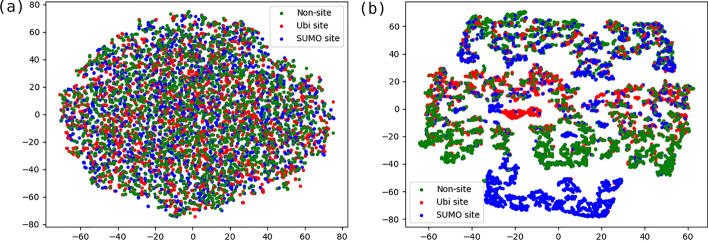
t-SNE visualization of **a** input layers and **b** merged layer

The graphical sequence logo was generated by the WebLogo tool to visualize amino acid residue conservation at a given position as Fig. 5. Amino acid residues Glu (E), Glu(G), Lys(K), Leu (L) appeared more frequently in positive samples of Ubiquitylation fragments, while Glu (E), Leu (L), Pro(P), Arg(R) were more enriched in positive samples of SUMOylation fragments. The results indicated the dependencies of upstream and downstream amino acid sites, which is consistent with the article of Chen et al. [27].

t-SNE [28] plot was employed to visualize the discriminating ability of the raw inputs and merged deep representations from three classes as Fig. 6 shown. Different colors represented different classes. It clearly showed that the distributions of original features were disordered and messy. After mappings of multiple hidden layers, the sample distribution tended to separate, which implied that our multi-label classification model may detect distinguishing representations and fuse seven subnets to further enhance the discriminative ability of our model. But in the meanwhile, some overlaps resulting in not complete distinctive boundaries also can be observed from the t-SNE plot. We reasoned such heterogeneous samples located closely at feature space implied crosstalk sites and some potential unlabeled positive samples. We investigated the crosstalk sites from our experimental data and found they accounted for nearly 2% out of total Ubiquitylation and SUMOylation sites. Such crosstalk samples reflected characteristics of Ubiquitylation and SUMOylation sites and were marked either Ubiquitylation sites (in red) or SUMOylation sites (in blue) in Fig. 6. Therefore, the overlaps between red and blue dots likely represented the crosstalk sites. Almost negatives (in green) in Fig. 6 were concentrated in a region except a small part scattered at the zones enriched in red dots and blue dots.

### Compared with dimensional reduction using PCA

**Fig. 7:**
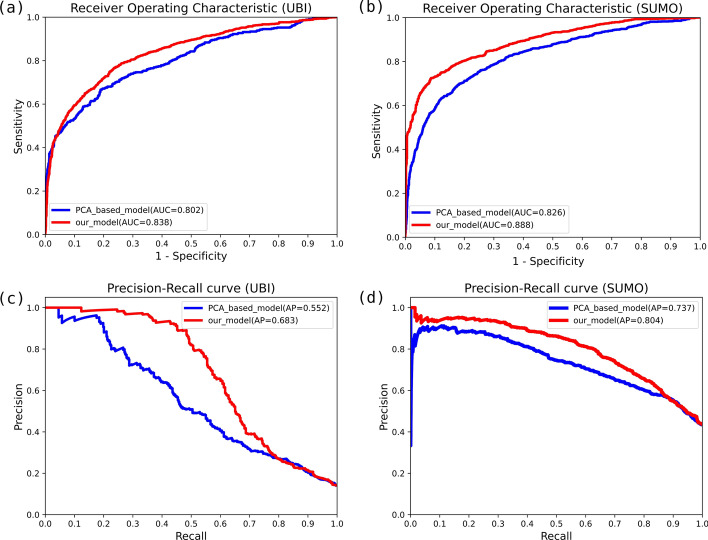
Comparisons between our model and PCA model. The performance of the multi-label model with ROC curves on **a** Ubiquitylation site prediction and **b** SUMOylation site prediction, and PR curves on **c** Ubiquitylation site prediction, and **d** SUMOylation site prediction. In each panel, the red line indicates the classification results were computed by our model while the blue line is the results based on the PCA of physical and chemical properties in Mathura’s research. The red line in the figure was higher than the blue line, which showed that our model can obtain more potential associations of features and better classification results

Principal component analysis (PCA) is a popular feature selection method that conducts a linear transformation to convert the original variables to a set of new orthogonal variables [29], which enables to avoid manual feature selection. Venkatarajan et al. reduced the multidimensional scaling of 237 physicochemical properties to 5-dimensional representations by using PCA [30]. We used this method to reduce the dimension from 402 to 6 as the original input, to include the principle componenets of physical-chemical properties for comparisions (Fig. 7).

### Independent dataset results

**Fig. 8:**
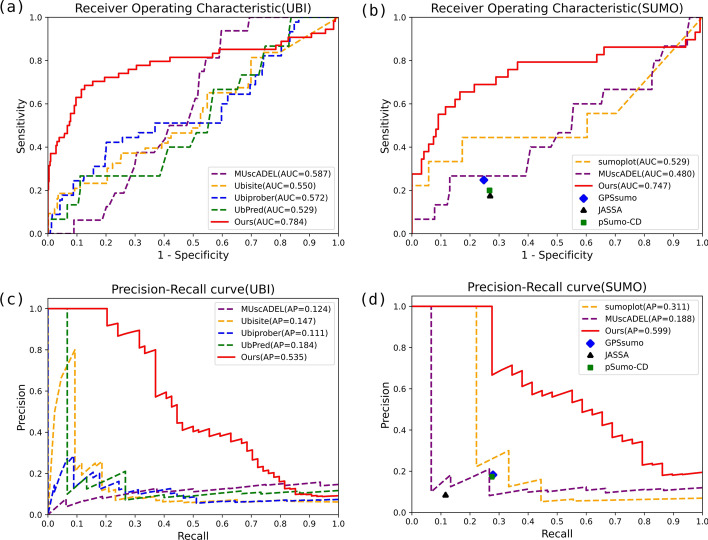
Comparisons to other methods on the independent test set ROC curves on **a** ubiquitylation site prediction and **b** SUMOylation site prediction, and PR curves on **c** ubiquitylation site prediction, and **d** SUMOylation site prediction

For fair evaluations, we built another independent set whose protein sequences were collected from UniProt/Swiss-Prot database updated after November 2020. At that point we had completed our data collection and all other tools had been published. Thereby, this dataset never appeared in our tool nor others, and served as an independent comparison. The details of this newly collected dataset are summarized in Additional file 1: Table S1. The performance of our method and others on the independent set is shown in Fig. 8. We also obtained an AUC and AP of 0.765 and 0.441 on crosstalk site prediction respectively.

## Discussion

In our pipeline, ensemble learning is used to simultaneously identify protein Ubiquitylation sites and SUMOylation sites as well as their crosstalk sites. Different from common ensemble approaches of using a simple average or predefined weights, our ensemble subnet enabled learning combined weights in a data-driven fashion.The led us to outperform other meta classifiers on both Ubiquitylation site prediction and SUMOylation site prediction.

Since we used the ensemble layer to synthesize two types of input features, the model can adaptively learn effective features. In addition, two PTMs mutually supported and boosted the multi-label prediction performance. Because crosstalk cannot be positive in both categories for ROC and PR curves, we calculated its AUC and AP of 0.862 and 0.552 respectively.

We also explored overlapping samples with similar deep features in t-SME to Ubiquitylation sites and/or SUMOylation sites that were potentially unrevealed positive sites, since only a small fraction of protein post-translational modification (PTM) sites were experimentally annotated in the records from Swiss-Prot [31].

The comparative results of PCA showed our deep architecture enables to generate more informative representations. As shown in Fig. 7, our model can obtain more potential associations between features and better classification results.

Our tool showed stronger discerning power than all other listed tools on the unseen samples in terms of AUC and AP. This can be attributed in part to adaptively integrating richer input modalities with seven descriptors and applying bootstrapping strategy to balance positive and negative samples in modeling.

## Methods

### Benchmark dataset

We built a benchmark dataset by collecting annotations from UniProt/Swiss-Prot (Nov 2020 release) [32]. This database provides high-quality protein sequences and manual annotations, including the descriptions of amino acid residue modification. To avoid the overestimation of model performance caused by homogenous sequences, we used the Cluster Database at High Identity with Tolerance (CD-HIT) [33] to remove proteins that have more than 40$\%$ sequence identity. Afterwards, 1983 proteins of Ubiquitylation and 4728 proteins of SUMOylation remained. Experimentally validated lysine (K) residues, based on the annotation from UniProt/Swiss-Prot, were taken as positive samples. The rest of the lysine (K) residues in the proteins were regarded as negative samples. In total, we obtained 4222 ubiquitylated sites, 56544 non-ubiquitylated sites, 16432 SUMOylated sites, 203533 non-SUMOylated sites. Then, we retrieved the crosstalk sites from Swiss-Prot database by the keyword ‘cross-link’. Through this search, we collected 401 crosstalk sites for our basic datasets. We organized all details of the datasets including the number of Ubiquitylation and SUMOylation sites, and the ratio between positive and negative samples into the Additional file 2: Table S2. For the further details of the involved proteins, we saved all sequence names and fragments of the retrieved proteins in Additional file 3: Tables S3 and Additional file 4: Table S4 respectively.

In this study, we employed tenfold cross-validation to evaluate the performance of the model. In this process, all the proteins of Ubiquitylation and SUMOylation were partitioned into 10 equal parts. The ratio of the training, validation and testing sets was 8:1:1. The details of the tenfold cross-validation dataset and the independent testing set are listed in the Additional file 1: Table S1 and Additional file 2: Table S2.

According to our previous grid search, a sliding window with a length of 24$\times$2+1=49 to intercept the protein sequence containing lysine residues (K) in the middle was optimal to deliver robust Ubiquitylation site prediction. The same settings were applied in this study consider the similarity between protein Ubiquitylation and SUMOylation. The details of optimized window size is explored in our previous work [34]. If the number of upstream and downstream positions was less than 24, then the placeholder was used to supplement. Moreover, the identifier “X” (unknown) was used to represent amino acids in the sequence that was not recognized by current sequencing techniques. “X” will be assigned an average value of 20 amino acids.

### Encoding of protein fragments

The following two types of encoding were adopted to encode the amino acid composition of the original protein fragment [35].

The first encoding converted 20 amino acids and one placeholder to a binary feature matrix. The corresponding state of the amino acids on each vector was 1, and the remaining indeices were 0. All of the 49-length amino acid fragments were then organized as a matrix of size 49*21.

We also utilize physical-chemical properties (PCPs) encoding, which can be found and downloaded from the AAindex database [36]. In this study, all physical-chemical properties were divided into six highly correlated clusters. Then, each sequence fragment was coded into 6 two-dimensional (2D) matrices. The details of six physical-chemical properties are shown in Table 1.

### Deep learning architecture

Our deep learning architecture consists of seven subnets to handle seven input modalities (one-hot and six physical-chemical properties encoding matrices). The structure and detailed hyper-parameters of these subnets can be found in Table 1.

**Table 1:** Hyper-parameters of proposed deep architecture

Subnets	Layer category	Hyper-parameters			
		Activation function	Size	Filters	Dropout
Sequence	1D Convolution	Relu	2	201	0.4
		Relu	3	151	0.4
		Relu	5	101	0.4
	Dense	Relu	256	–	0.3
		Relu	128	–	0
		Sigmoid	2	–	–
Physico-O	Dense	Relu	256	–	0.2
		Relu	128	–	0.1
		Sigmoid	2	–	–
Physico-P	Dense	Relu	512	–	0.3
		Relu	256	–	0.2
		Relu	128	–	0.1
		Sigmoid	2	–	–
Physico-H	Dense	Relu	1024	–	0.4
		Relu	512	–	0.3
		Relu	256	–	0.2
		Relu	128	–	0.1
		Sigmoid	2	–	–
Physico-C	1D Convolution	Relu	2	201	0.2
		Relu	3	151	0.1
	Dense	Sigmoid	2	–	–
Physico-B	1D Convolution	Relu	2	201	0.3
		Relu	3	151	0.2
		Relu	5	101	0.1
	Dense	Sigmoid	2	–	–
Physico-A	1D Convolution	Relu	2	201	0.4
		Relu	3	151	0.3
		Relu	5	101	0.2
		Relu	7	51	0.1
	Dense	Sigmoid	2	–	–
Ensemble	Dense	Relu	7	–	–
		Sigmoid	2	–	–

The first subnet was designed to extract the internal correlation between adjacent amino acids, and highlight the meaningful part of the feature maps. Thereafter, we merged all the newly generated feature maps with three dense layers, to produce a low dimensional feature representation. The rest of the subnets would each take one of the six groups of the physical-chemical properties defined by Tomii et al. in the AAindex database [36]. These included alpha and turn propensities, beta propensity, composition, hydrophobicity, physicochemical properties, and other properties. According to the dimension of the input feature matrices, the layers and hyperparameters of the network structure were adjusted accordingly.

These subnets separately detected intra-correlations and generated deep representations for each group of physical-chemical properties. Baseline experiments were conducted by training convolution layers and fully-connected layer with sigmoid activation function and nesterov adaptive moment estimation (Nadam) optimization algorithm, and a categorical cross-entropy loss function [37]. Since the number of samples between each class and negatives were imbalanced, a class-weight was set inversely proportional to the number of samples in the class to equalize the contribution of each class and the negatives. The class weights were calculated as follows:

1 $$\begin{aligned} \begin{aligned} v_i = {{1\over c_i}\over {avg(\sum _{i=0}^{n}{1\over c_i}})} \end{aligned} \end{aligned}$$

The number of samples for each class is represented by *n* and *C*$_{i}$ represents the weight of the class *i.* category. And binary cross-entropy [37] was used as the loss function to drive model fitting. The output layer independently maps the embedding from previous layers to generate two probabilities for Ubiquitylation and SUMOylation via the sigmoid function. All models were respectively trained using a maximum of 100 epochs and stopped early if there was no further improvement in loss for any 10 continuous epochs.

### Ensemble learning

Our deep learning predictor incorporated an ensemble learning strategy, to predict protein Ubiquitylation sites and SUMOylation sites precisely and conveniently as well as their crosstalk sites. The seven well-trained subnets can be considered as seven meta classifiers for the parallel completion for the prediction task [38]. We included an additional fully connected layer to integrate the outputs from the seven subnets. Such stacking-based ensemble learning enables us to adaptively coordinate inter-class diverse meta-learners and generate better predictions [39, 40]. When training the whole ensemble network, we loaded the pretrained weights of all layers before the logits of each meta subnet respectively, and carried out the training procedure with the same training settings.

Since only a small number of lysine post-translational modification sites occur in protein sequences, the distribution of positive and negative samples is extremely imbalanced. Therefore, we employed bootstrapping for resampling the training data.This can help generate a more stable and unbiased model. Assuming that *pos* and *neg* represented the number of positive samples and negative samples, bootstrapping randomly selected *pos* positive samples and *neg* negative samples during the sampling process to form a balanced training set. Therefore, the network can be trained *N* (*N*= the number of negatives / the number of positives) times to learn the weight. In this study, by randomly resampling the negative samples in equal proportionto the positive number, we were able to balance positive samples (including Ubiquitylation sites and SUMOylation sites) and negative samples involved in each training iteration. According to the distribution between positives and negatives, as seen in Additional file 2: Table S2, such boostrapping procedure would go through 13 iterations in a training epoch to take as many negatives as possible in training. The specific data points from each class after balanced are described in Additional file 5: Table S5.

## Conclusion

In this paper, we proposed a novel ensemble deep learning based predictor for simultaneously identifying protein Ubiquitylation sites and SUMOylation sites as well as their crosstalk sites. Overall, the highlight of our method is mainly due to its data-driven feature, multi-label formulation for Ubiquitylation and SUMOylation sites, and ensemble learning. Because of the natural structural and functional similarity between Ubiquitylation and SUMOylation, the data regarding two PTMs supported each other and boosted the multi-label prediction performance. The designed ensemble learning layer synthesized the results of multiple meta classifiers and avoided the possibility of poor generalization performance due to a single classifier.

By comparing with the results of similar tools, our ROC curves and PR curves were stable at a higher level. This demonstrated the effectiveness of our method and robustness of the ensemble models, and reflected the potential of the deep learning algorithm in the field of Ubiquitylation and SUMOylation protein sites prediction. Because the input of our architecture is not particularly designed for Ubiquitylation and SUMOylation, our architecture can extend to other PTMs easily without any adjustment. Further research will explore incorporating newly updated Ubiquitylation and SUMOlytion data to incremenetally upgrade our model, and extending our architecture to other types of PTMs.

## Supplementary information

**Additional file 1**.** Table S1**: Details of independent test set

**Additional file 2**.** Table S2**: Details of the dataset division of the 10-fold cross-validation

**Additional file 3**.** Table S3**: All sequence names of retrieved proteins

**Additional file 4**.** Table S4**: All fragments of retrieved proteins

**Additional file 5**.** Table S5**: Distribution of positive and negative samples in each iteration using bootstrapping

## Data Availability

The source code and the datasets used in this work are available at https://github.com/lijingyimm/MultiUbiSUMO.

## Abbreviations

ROC: Receiver operation characteristic
AUC: Area under the receiver operating characteristic curve
PR: Precision-Recall
AP: Average precision
SUMO: Small ubiquitin-like modifier
t-SNE: t-distributed stochastic neighbor embedding
PCA: Principal component analysis
