# Supplementary material for: An Ensemble Deep Learning based Predictor for Simultaneously Identifying Protein Ubiquitylation and SUMOylation Sites
Source: BMC Bioinformatics. 2021 Oct 24;22:519. doi: 10.1186/s12859-021-04445-5 (PMC8543953; doi:10.1186/s12859-021-04445-5)
Supplement: Supplementary file 1 — Additional file 1. Table S1: Details of independent test set [file 12859_2021_4445_MOESM1_ESM.docx]

Table 1. Detail of independent test set

| Dataset  The type of site | The number of protein sequences | Number of positive data | | | Number of negative data | The ratio between positive and negative samples |
| --- | --- | --- | --- | --- | --- | --- |
|  |  | Ubiquitylation | SUMOylation | crosstalk | Non-site |  |
| independent set | 23 | 48 | 13 | 7 | 721 | 1:11.8 |
