# Supplementary material for: An Ensemble Deep Learning based Predictor for Simultaneously Identifying Protein Ubiquitylation and SUMOylation Sites
Source: BMC Bioinformatics. 2021 Oct 24;22:519. doi: 10.1186/s12859-021-04445-5 (PMC8543953; doi:10.1186/s12859-021-04445-5)
Supplement: Supplementary file 2 — Additional file 2. Table S2: Details of the dataset division of the 10-fold cross-validation [file 12859_2021_4445_MOESM2_ESM.docx]

Table 2. Details of the dataset division of the 10-fold cross-validation

| Dataset | | The number of protein sequences | Number of positive data | | | Number of negative data | The ratio between positive and negative samples |
| --- | --- | --- | --- | --- | --- | --- | --- |
| 10 fold | The type of site |  | Ubiquitylation | SUMOylation | crosstalk | Non-site |  |
| 1 | Training | 5368 | 3359 | 13104 | 308 | 208422 | 1:12.7 |
|  | Validation | 671 | 428 | 1651 | 38 | 26284 | 1:12.6 |
|  | Testing | 672 | 435 | 1677 | 55 | 25371 | 1:12.0 |
| 2 | Training | 5364 | 3389 | 12745 | 307 | 208920 | 1:12.9 |
|  | Validation | 673 | 423 | 1683 | 38 | 26482 | 1:12.6 |
|  | Testing | 674 | 433 | 1645 | 56 | 25326 | 1:12.2 |
| 3 | Training | 5367 | 3388 | 13563 | 308 | 207864 | 1:12.3 |
|  | Validation | 672 | 422 | 1645 | 37 | 26468 | 1:12.8 |
|  | Testing | 672 | 431 | 1669 | 56 | 25223 | 1:12.0 |
| 4 | Training | 5366 | 3393 | 13537 | 309 | 208731 | 1:12.3 |
|  | Validation | 673 | 427 | 1692 | 37 | 26189 | 1:12.4 |
|  | Testing | 672 | 441 | 1699 | 55 | 25345 | 1:11.8 |
| 5 | Training | 5369 | 3422 | 12638 | 300 | 208409 | 1:13.0 |
|  | Validation | 671 | 423 | 1614 | 38 | 26315 | 1:12.9 |
|  | Testing | 671 | 431 | 1665 | 63 | 25177 | 1:12.0 |
| 6 | Training | 5367 | 3305 | 13102 | 316 | 208230 | 1:12.7 |
|  | Validation | 672 | 428 | 1615 | 39 | 26332 | 1:12.9 |
|  | Testing | 672 | 434 | 1661 | 44 | 25375 | 1:12.1 |
| 7 | Training | 5365 | 3370 | 12827 | 312 | 208128 | 1:12.8 |
|  | Validation | 673 | 425 | 1652 | 37 | 26114 | 1:12.6 |
|  | Testing | 673 | 439 | 1679 | 52 | 25546 | 1:12.1 |
| 8 | Training | 5370 | 3395 | 13357 | 304 | 208503 | 1:12.4 |
|  | Validation | 671 | 426 | 1605 | 39 | 26461 | 1:13.0 |
|  | Testing | 670 | 440 | 1681 | 58 | 25405 | 1:12.0 |
| 9 | Training | 5368 | 3325 | 13258 | 310 | 208719 | 1:12.6 |
|  | Validation | 672 | 427 | 1649 | 38 | 26444 | 1:12.7 |
|  | Testing | 671 | 430 | 1652 | 53 | 25239 | 1:12.1 |
| 10 | Training | 5367 | 3349 | 12715 | 304 | 208294 | 1:13.0 |
|  | Validation | 671 | 426 | 1619 | 39 | 26571 | 1:13.0 |
|  | Testing | 673 | 431 | 1671 | 58 | 25540 | 1:12.2 |
