# Supplementary material for: An Ensemble Deep Learning based Predictor for Simultaneously Identifying Protein Ubiquitylation and SUMOylation Sites
Source: BMC Bioinformatics. 2021 Oct 24;22:519. doi: 10.1186/s12859-021-04445-5 (PMC8543953; doi:10.1186/s12859-021-04445-5)
Supplement: Supplementary file 5 — Additional file 5. Table S5: Distribution of positive and negative samples in each iteration using bootstrapping [file 12859_2021_4445_MOESM5_ESM.docx]

Table 5. Distribution of positive and negative samples in each iteration using bootstrapping

| 10-fold | Number of positive samples | | Number of negative samples per iteration |
| --- | --- | --- | --- |
|  | Ubiquitination | SUMOylation |  |
| 1 | 3359 | 13104 | 16463 out of 208422 |
| 2 | 3389 | 12745 | 16134 out of 208920 |
| 3 | 3388 | 13563 | 16951 out of 207864 |
| 4 | 3393 | 13537 | 16930 out of 208731 |
| 5 | 3422 | 12638 | 16060 out of 208409 |
| 6 | 3305 | 13102 | 16407 out of 208230 |
| 7 | 3370 | 12827 | 16197 out of 208128 |
| 8 | 3395 | 13357 | 16752 out of 208503 |
| 9 | 3325 | 13258 | 16583 out of 208719 |
| 10 | 3349 | 12715 | 16064 out of 208294 |
